# Infectious Keratitis: Secreted Bacterial Proteins That Mediate Corneal Damage

**DOI:** 10.1155/2013/369094

**Published:** 2013-01-08

**Authors:** Mary E. Marquart, Richard J. O'Callaghan

**Affiliations:** Department of Microbiology, University of Mississippi Medical Center, 2500 N. State Street, Jackson, MS 39216, USA

## Abstract

Ocular bacterial infections are universally treated with antibiotics, which can eliminate the organism but cannot reverse the damage caused by bacterial products already present. The three very common causes of bacterial keratitis—*Pseudomonas aeruginosa*, *Staphylococcus aureus*, and *Streptococcus pneumoniae*—all produce proteins that directly or indirectly cause damage to the cornea that can result in reduced vision despite antibiotic treatment. Most, but not all, of these proteins are secreted toxins and enzymes that mediate host cell death, degradation of stromal collagen, cleavage of host cell surface molecules, or induction of a damaging inflammatory response. Studies of these bacterial pathogens have determined the proteins of interest that could be targets for future therapeutic options for decreasing corneal damage.

## 1. Introduction

The bacterial agents of infectious keratitis that have been studied in considerable detail are three of the most common causes of such infections, namely, *Staphylococcus aureus*, *Streptococcus pneumoniae*, and *Pseudomonas aeruginosa* [1]. The mechanisms underlying the tissue damage occurring during these infections have been studied in animal models. These infections are initiated by injection of bacteria into the corneal stroma, usually of New Zealand rabbits, or by the application of a topical drop of bacteria to a scarified cornea, usually of a mouse [2]. Important to this research is the relative virulence of three forms of a bacterial strain, namely, the unaltered parent strain, its mutant deficient in a single specific gene coding for a secreted protein, and that same mutant strain following insertion of a functional copy of the mutated gene, a rescued strain. If the parent and rescue strains have statistically equivalent virulence and the mutant has significantly less virulence, then the mutated gene is recognized as a key virulence factor for the cornea [3]. An additional method for establishing a specific gene as a virulence factor is to demonstrate that insertion of this specific gene into a nonpathogenic strain can significantly increase the virulence [3]. These types of genetic analysis of virulence have defined multiple virulence factors for each of the three organisms commonly causing keratitis.

The importance of secreted proteins to keratitis can be illustrated by the study of certain nonpathogenic strains of bacteria. One observation that is not generally recognized, but is very important to consider, is that bacteria can be injected into a rabbit cornea and there grow from a small inoculum to millions of bacteria without causing any harm to the eye [4, 5]. For instance, *Pseudomonas putida* has been shown to grow well without mediating inflammation or corneal damage. This organism has LPS and other surface molecules, but it does not secrete proteins with corneal toxicity. This harmless infection is unlike that seen in an infection with the same *P. putida* strain after it has been modified by the insertion of a plasmid bearing a single *P. aeruginosa* gene coding for a secreted protease known to be a corneal virulence factor [4]. In fact, the secretion of any one of the three known *P. aeruginosa* proteases can result in a virulent infection [5].

The value of knowing the mechanisms of bacterial corneal virulence relates to the need to limit such mechanisms before the tissue damage deprives the eye of vision. Application of an antibiotic to an infected eye can eliminate the infecting bacteria, but the damaging bacterial proteins already secreted can continue to mediate harmful inflammation and act directly to damage the cornea [6]. The inclusion of a steroid during antibiotic therapy helps control the inflammatory process, but the actions of the secreted proteins are not affected by such therapy [7, 8]. Knowledge of the key mediators of tissue damage must be known to allow subsequent development of adjunct therapies to limit the action of these bacterial proteins. The prospect of using the immune system to inhibit these secreted bacterial proteins has a merit, but the bacterial enzymes found to be active in damaging corneal tissue may be poor immunogens or the antibody produced may not be effective in impeding the enzymatic activity [9]. Thus, the mechanisms of keratitis have partially evaded the benefit of our current therapies. Also problematic is the emergence of bacteria with greater resistance to those antibiotics that were highly successful for many previous years; delays in obtaining an effective therapy provides time for the bacterial population to expand and to continue secreting the damaging proteins. 

## 2. **Pseudomonas aeruginosa**

*Pseudomonas* corneal infections typically are associated with the use of contact lenses; that is, this is a man-made disease which was rarely a problem during the centuries prior to the contact lens use [10–12]. The organism, seen as a single gram-negative rod, is found in the environment, especially in moist places, so it is often available to contaminate the contact lens cases [10]. Its adherence to plastic, coupled with its resistance to disinfectants, favors its introduction into the eye. These organisms can react with a corneal defect in the epithelium and they can pass through the epithelial barrier to the corneal stroma [13]. Once these organisms reach the corneal stroma, the infections can rapidly progress toward melting of the cornea, an event attributed to the bacterial proteases, the activation of matrix metalloproteinases, and a damaging immune response that delivers among other things both reactive oxygen intermediates and host proteases [5]. 

*P. aeruginosa* is capable of secreting at least seven different proteases; these are elastase A (Las A), elastase B (Las B) [14], modified elastase [15], alkaline protease (AP), protease IV, pseudomonas aeruginosa small protease (PASP) [15], and the large exoprotease (Lep A) [16]. Las A, Las B, modified elastase, and AP are metalloproteinases and may be produced by only some strains [17]. These metalloproteases, especially Las B and AP, have been well studied in terms of their potential contribution to keratitis. These enzymes, especially Las B, upon injection into the corneal stroma can mediate considerable corneal damage [18, 19]. However, mutation of any one of these genes does not result in significantly reduced virulence of the organism [20–22]. Also, data exist showing that a strain with the potential to produce Las B fails to produce the enzyme during keratitis [23]. *P. putida,* when supplied with a plasmid-borne gene for Las B, secretes Las B and demonstrates significant virulence in the infected rabbit cornea [5]. 

Lep A is a large protease that has been postulated to be a serine protease [16, 24]. The enzyme has been shown to be a virulence factor in a nonocular model of infection, but to date there are no published studies of its role in an ocular infection. An important study on Lep A showed that this enzyme can cleave a host transmembrane protein, designated the protease-activated receptor, which, when cleaved, can induce the production of cytokines [16]. This study helps explain the inflammation that results when the bacteria producing proteases interact with corneal cells. This finding may also clarify the failure of *P. putida* to cause inflammatory changes as it grows in the cornea whereas the same stain engineered to produce a *P. aeruginosa* protease causes significant inflammation. 

In contrast to the findings with the metalloproteases, the serine protease PIV is produced by essentially all strains able to cause keratitis [25, 26]. Mutation of the PIV gene has been shown to reduce the corneal virulence and the corneal virulence was restored following the insertion into the mutant strain of a plasmid coding for functional PIV [25]. When a PIV-coding plasmid was inserted into *P. putida*, the organism acquired a significant amount of corneal virulence [4]. PIV is able to cleave many proteins; in fact, few proteins with lysine escape cleavage by this protease [27, 28]. PIV will cleave poly-L-lysine to small peptides and free lysine. Cleaved by PIV are a variety of host defense proteins including immunoglobulins, complement components, antimicrobial peptides, and surfactants [27–29]. This protease contributes to virulence yet it is inefficient in cleaving collagens, the chief structural component of the corneal stroma. PIV has a molecular weight of 27,384 daltons and it can aggregate following exposure to SDS to enzymatically active masses of >200,000 daltons [27, 28]. Extensive immunizations with recombinant PIV were needed to produce even a relatively small amount of antibody to recombinant PIV or native PIV [9]. The antibodies that were produced failed to inhibit the PIV catalytic activity or to provide protection from corneal damage due to the injection of active enzyme [9]. The enzyme activity is susceptible to TLCK, a serine protease inhibitor, but unfortunately TLCK is highly toxic for eyes [28]. The development of a nontoxic inhibitor could be very useful as an adjunct therapy for treating these infections. 

The PASP protease has recently been analyzed relative to its role in keratitis and its properties as an enzyme [15, 30, 31]. The PASP gene is found in all strains tested and western blots of culture supernatants indicate that it is commonly expressed and secreted by both clinical isolates and established lab strains [30]. A mutant lacking the PASP gene was engineered and found to have significantly less virulence than its parent or its rescued strain [31]. The reduced virulence of the PASP-deficient mutant was demonstrated in both the rabbit intrastromal injection model and the mouse scratch model of keratitis. Injection of purified PASP into the rabbit cornea results in the destruction of the epithelium and the formation of erosions that can reach into the stroma [30]. PASP, like PIV, is not neutralized by antibody to the recombinant PASP protein and the antibody does not protect the cornea from the injected enzyme or active infection [30]. 

The native PASP protein as found in culture supernatants has a molecular weight of 18,500 daltons [15, 30]. Studies of the purified enzyme or its recombinant protein showed that, unlike PIV, PASP does not efficiently cleave many host proteins [15, 30]. However, PASP is able to cleave collagens and this trait further distinguishes PASP from PIV. Cleavage of collagen by PASP suggests that this enzyme could be important in the destruction of the cornea [30]. PASP will cleave peptides with lysine or arginine and it can convert poly-L-arginine or poly-L-lysine to small peptides and free amino acids. PASP appears to be a serine protease that is susceptible to serine protease inhibitors (e.g., TLCK) and partially inhibited, in a non-dose-dependent fashion, by high concentrations of EDTA (>100 mM). Enzymatic activity may require the formation of a homodimer in order to create the triad of three amino acids that form the active catalytic site [31].

The possibility exists that PIV provides the bacteria with a defense against multiple host defense molecules whereas PASP is active in cleaving the collagen-based structure of the cornea. Once a sizeable population of *P. aeruginosa* is established in a tissue site, a great deal of host response and tissue damage occurs before the infection is cleared. 

## 3. **Streptococcus pneumoniae**

 Recent attention has been given to *S. pneumoniae* (pneumococcus) as a major cause of conjunctivitis outbreaks; however, this organism is also a common cause of infectious keratitis. Some epidemiologic studies identify the pneumococcus as the top cause of bacterial keratitis [32–39]. Most other reports show this bacterium and other *Streptococcus* species to be the causative agents most commonly encountered after *P. aeruginosa* and/or *S. aureus* [40–49]. Pneumococcal keratitis is not typically contact lens associated like *P. aeruginosa*, but predisposing conditions such as ocular trauma or surgery are factors in this disease [32, 33, 36, 39, 41, 48, 50–54].

 The outer capsule of *S. pneumoniae*, while not protein but rather polysaccharide in composition, is likely the single most studied virulence factor of this bacterium. Pneumococcus normally resides in the human nasopharynx, and studies that date back to early bacterial virulence and transformation experiments such as those of Griffith [55] and Avery et al. [56] determined that the polysaccharide capsule (i.e., the characteristic that provides a “smooth” colony appearance) is the component necessary for *S. pneumoniae* to establish virulence and survive the host immune system. The central dogma of pneumococcal virulence in infections such as pneumonia, otitis media, meningitis, and septicemia is that noncapsular bacteria are avirulent. This long held rule was proven to be untrue for keratitis, as noncapsular strains were shown to cause as severe keratitis as their capsular counterparts in intrastromal infection models [57, 58].

 Other than the capsule, the pneumococcus possesses a variety of proteins that have been characterized as virulence factors in nonocular models of disease. One such protein is pneumolysin, which is a toxin belonging to the family of bacterial cholesterol-dependent cytolysins. Pneumolysin is a 53 kDa protein that does not possess any known secretion signal sequence. This cytolysin was long thought to be an intracellular protein released from the bacteria upon cell lysis [59–61] and more recently reported to be cell wall associated [62]. The mode of action of pneumolysin is the binding of monomers to cholesterol in host cell membranes, oligomerization at the membrane into 30–50 mers, and pore formation resulting in host cell lysis [63–65]. Lower concentrations of pneumolysin have been reported to stimulate leukocyte migration [66] and activate complement [67], thus stimulating the host inflammatory response and causing immunomediated damage to host tissues. Johnson and Allen first identified pneumolysin as responsible for ocular tissue damage during pneumococcal keratitis and characterized the biochemical features *in vitro* and the role in corneal virulence *in vivo* [68, 69]. Pneumolysin was verified to play a major role in keratitis as evidenced by reduced corneal virulence of a pneumolysin-deficient strain of *S. pneumoniae* compared to its parent strain in a rabbit intrastromal infection model [70]. Mutation of the complement activation domain of pneumolysin also resulted in decreased corneal virulence, particularly inflammation [71]. Added to these findings was that induction of leukopenia in rabbits resulted in significantly decreased severity of corneal damage following challenge with purified pneumolysin, indicating that pneumolysin was at least partly responsible for triggering the inflammatory response and causing immunomediated damage [72]. Moreover, these and more recent studies have shown that pneumococcal keratitis continues to worsen in animal models when the bacteria have either reached a very low number or have been completely cleared [58, 71, 73, 74], underscoring the fact that corneal damage occurs despite eradication of the bacteria by antibiotics. 

Efforts to find feasible pneumolysin inhibitors for the cornea have led to the use of soluble cholesterol as a topical therapy. Cholesterol was long known to be effective in inhibiting the hemolytic activity of pneumolysin *in vitro* [75], so this concept was employed *in vivo*. Topical cholesterol was shown to significantly reduce corneal inflammation in pneumococcal keratitis, purportedly by acting as a competitor for host cholesterol and neutralizing pneumolysin [76, 77]. Another attempt at targeting pneumolysin was the use of active and passive immunization in an animal model of keratitis [73, 78].

Proteins other than pneumolysin have been shown to be important for virulence in nonocular pneumococcal diseases but have not been associated with keratitis to date. Most of these proteins are located on the bacterial cell surface. A detailed review of pneumococcal virulence proteins is described elsewhere [79]. Choline-binding proteins such as pneumococcal surface proteins A and C (PspA and PspC) are anchored by their bonds to choline in the cell wall. Other proteins are those containing LPXTG motifs that are recognized by bacterial sortase enzymes and are placed on the cell surface. One pneumococcal LPXTG protein of interest is neuraminidase A (NanA), which cleaves N-acetylneuraminic acid from host cell components and is suggested to be important for the binding of pneumococci to conjunctival epithelial cells by degrading host cell mucin [80]. A variety of surface-associated proteins that do not fall into the categories of choline-binding proteins or LPXTG proteins have also been identified as involved in adherence, immune evasion, immune activation, and enzymatic reactions in nonocular models. Finally, at least four zinc metalloproteinases, including an IgA protease, have been identified with conflicting reports of their cellular locations. One of these proteases, ZmpC, has recently been shown to induce ectodomain shedding of a membrane-associated mucin from cultured conjunctival and corneal epithelial cells [81]. Much remains unknown, however, as to the factors other than pneumolysin that contribute to pneumococcal keratitis.

## 4. **Staphylococcus aureus**

*S. aureus* is the most common cause of bacterial keratitis and an important cause of other ocular infections [82–87]. This gram-positive coccus is found in human carriers who retain the organism in their anterior nares, throat, and perianal body sites [88]. Specific strains of bacteria found in the flora around the eye provide the source of organisms that infect the eye [89]. Humans are one reservoir for this organism, and multiple animals, especially pigs, have had an important role in the epidemiology of *S. aureus *[90]. Keratitis occurs in individuals whose eyes are compromised by any of multiple changes including ocular surgery, contact lens use, trauma, viral infection, or other illnesses [91–93]. *S. aureus* is well known for its ability to evolve mechanisms of antibiotic resistance, making these infections among the most difficult to treat, and antibiotic resistance has increased since 2000 [94–97]. Although the antibiotic resistance of *S. aureus* is well known, what is less well recognized is that the gene transfer mechanisms that created the highly resistant strains can also transfer virulence traits [98–100]. Bacteriophage provides horizontal transfer of individual genes as well as clusters of virulence traits in a genetic unit designated as a pathogenicity island [98]. Individual strains can develop a set of genes that allow their emergence as a cause of life-threatening infections. 

The mechanisms involved in the initiation of keratitis are not yet understood. *S. aureus* has been shown to bind to human corneal cells in culture, a reaction mediated by the fibronectin-binding protein on the bacterial surface [101]. Binding to the cornea can also be mediated by a collagen-binding adhesin on the *S. aureus* surface [102]. Despite this binding ability and the availability of organisms from the flora surrounding the eye, keratitis infrequently develops. Possibly the greatest barrier protecting the eye is the bactericidal enzyme phospholipase A_2_, a component of the tear film [103, 104]. Once inflammation occurs in the anterior portion of the eye, the amount of phospholipase A_2_ increases, thus enhancing protection against bacteria [104]. The topical application of *S. aureus* to a scarified rabbit cornea typically fails to cause an infection and results in the rapid loss of the bacteria [104]. An active infection of the rabbit cornea has been achieved by treating a bacterial inoculum on a contact lens with spermidine and applying spermidine to the eye both before and after applying the contaminated lens to the corneal surface [105]. Spermidine is able to bind to the bacterial surface and prevent bacterial killing by phospholipase A_2_. There is, however, one unique strain of *S. aureus* that can infect the scarified cornea without any other treatments; this strain is susceptible to the bactericidal action of tears but has demonstrated an enhanced invasion of human corneal epithelial cells *in vitro* [unpublished finding]. These data suggest that keratitis is dependent on bacterial binding and rapid penetration of the corneal epithelium. 

The events occurring once bacteria reach the corneal stroma have been studied in some detail. The virulence of a prototype *S. aureus* strain (8325-4) following its injection into the rabbit cornea was significantly reduced by a mutation of the gene coding for alpha-toxin, a lytic cytotoxin produced by nearly all *S. aureus* strains [106]. The alpha-toxin rescue strain had corneal virulence equivalent to that of the parent strain. Nanogram quantities of purified alpha-toxin were shown to cause extensive sloughing of the corneal epithelium, corneal edema, and severe iritis [107]. The importance of this toxin was found also in the infection of the mouse cornea [108, 109]. Alpha-toxin is now recognized as the critical virulence factor in *S. aureus* pneumonia in humans [110]. Research on the alpha-toxin's mechanism of action indicates that the toxin enters the cytoplasmic membrane and moves laterally until seven subunits unite into a circular arrangement forming a pore in the cytoplasmic membrane [111]. The individual toxin molecules are thought to interact with caveolin-1 in the membrane to form a pore [111–113]. Inhibitors of the alpha-toxin-mediated lysis of erythrocytes have been developed by inserting lipid molecules into a cyclodextrin ring [114, 115]. One such inhibitor containing cholesterol has been shown to reduce the virulence of infections of the rabbit cornea [114]. As was described above, the cyclodextrin-based inhibitor active against alpha-toxin is also active against pneumolysin [76]. 

Alpha-toxin is not the only virulence factor contributing to the tissue damage during *S. aureus* keratitis; important also is gamma-toxin, a two-component toxin produced by nearly all *S. aureus* strains [116, 117]. Deletion of the gamma-toxin genes from a prototype strain, strain Newman, resulted in a significant loss in corneal virulence and restoration of these genes enhanced the virulence back to that produced by the parent strain [118]. A mutation in gamma-toxin or inhibition of this toxin has also been shown to reduce the virulence of endophthalmitis caused by *S. aureus* [119, 120]. Gamma-toxin is composed of an F component and an S component that are each nontoxic when tested alone [121, 122]. The S component is thought to bind to the target cell and only then will the F component bind [122]. The combination of F and S can move laterally in the cell membrane and multiple F-S pairs can combine into a ring that penetrates the membrane causing cell lysis [122–124]. A cyclodextrin-based inhibitor for the gamma-toxin has very recently been identified as an inhibitor of the gamma-toxin-mediated lysis of erythrocytes, but it has not been tested yet in infected corneas [120]. 

Gamma-toxin is only one of several two-component toxins produced by *S. aureus* and each has its own F and S components [122]. Gamma-toxin is composed of two proteins, either HlgA and HlgB or HlgC and HlgB; likewise the Panton-Valentine leucocidin (PVL), a well-recognized toxin, is composed of its two proteins, LukF-PV and LukS-PV [122]. Lesser known proteins involved in toxic activity include three S-type proteins LukM, LukS-R, and Luk E and two F-type proteins LukF-R and Luk D [122–124]. There is about 60–70% sequence homology among the various F components and a fairly similar degree of homology among the multiple S components [122]. The two-component toxin systems of *S. aureus* are complicated by the fact that the F component of one toxin can bind to the S component of another toxin [122]. Therefore, multiple unique toxins can be formed by one component from each of two different toxins (e.g., the F of one toxin with the S of another toxin). Each combination of an F and an S component can have its own specific toxicity [122]. How these toxins relate to corneal damage is yet to be resolved, but because these toxins are homologous with gamma-toxin, it is likely that at least one such toxin has corneal toxicity. 

Another molecule with proven ability to serve as a corneal virulence factor is the setnm-1 gene [125]. This gene is a part of a cluster of genes for super-antigen-like proteins involved in virulence for infections in nonocular sites [126]. These genes are found in a pathogenicity island on the chromosome of many *S. aureus* isolates and they are thought to have been derived from a phage genome [126]. The genes in this island, other than setnm-1, are known to inhibit host immune mediators such as complement or IgA. Only setnm-1 has been shown to mediate corneal damage; that is, a mutant deficient in this gene has significantly reduced corneal virulence as compared to its parent and rescued strains [125]. It is not yet clear how setnm-1 functions as a virulence factor, but one possibility is that it has protease activity [127]. Injection of the protein produced by the setnm-1 gene can cause extensive corneal damage, an event not expected for a super-antigen-like protein. 

## 5. Conclusions

The fundamental treatment of bacterial keratitis is based on the action of bactericidal antibiotic therapy to eliminate the bacteria that secrete the toxic proteins. A key issue in treatment is that the cornea has rapidly replicating bacteria prior to the onset of severe symptoms, and later, when symptoms are present, bacteria with a much reduced rate of bacterial replication predominate. Key drugs, such as fluoroquinolones, are far more effective on replicating than nonreplicating bacteria, so the treatment of the cornea needs to be prompt, aggressive, and prolonged. Global regulation of gene expression in bacteria limits the production of important toxins and enzymes until the bacteria are no longer rapidly replicating [128]. Once replication slows or stops, the toxins are secreted; thus, nonreplicating bacteria are hard to kill and they are efficient toxin producers. The points to be stressed are that prompt bactericidal therapy is a must and that the toxins once produced cannot now be eliminated without a loss of healthy tissue.
